# 2066 Functional characterization of mutant BRCA1

**DOI:** 10.1017/cts.2018.77

**Published:** 2018-11-21

**Authors:** John Barrows, David Long

**Affiliations:** University of South Carolina

## Abstract

OBJECTIVES/SPECIFIC AIMS: The objective of this work is to determine the mechanistic consequences of BRCA1 mutants in inter-strand crosslink (ICL) repair. METHODS/STUDY POPULATION: Our lab uses Xenopus egg extracts to study ICL repair. These extracts can be depleted of endogenous BRCA1 by immunoprecipitation. The goal of this work is to rescue endogenous depletion with in vitro translated, wild type BRCA1. Once achieved, we can supplement the depleted extract with BRCA1 mutants to access their function in ICL repair. RESULTS/ANTICIPATED RESULTS: We hypothesize that the BRCT and RING domain mutations will abrogate ICL repair, while mutations in the coiled coil region will not affect repair. DISCUSSION/SIGNIFICANCE OF IMPACT: These findings will have an immense impact on the understanding of BRCA1 domains. Importantly these results will spur personalized therapy of BRCA1 mutants by showing which domains are sensitive to cross-linking agents.

